# Inhibin/activin expression in human and rodent liver: subunits *α* and *β*B as new players in human hepatocellular carcinoma?

**DOI:** 10.1038/bjc.2011.53

**Published:** 2011-03-15

**Authors:** K Frost, K Seir, A Lackner, M Grusch, B Grasl-Kraupp, R Schulte-Hermann, C Rodgarkia-Dara

**Affiliations:** 1 Research Unit Chemical Safety and Cancer Prevention, Department Institute of Cancer Research, Clinics of Internal Medicine I, Medical University of Vienna, Borschkegasse 8A, Vienna 1090, Austria

**Keywords:** inhibin, activin, hepatocellular carcinoma, real-time PCR

## Abstract

**Background:**

Activins and inhibins belong to the TGF*β*-superfamily, which controls cell proliferation and differentiation in many organs. Activin A, the dimer of inhibin *β*A subunit, acts strongly anti-proliferative in hepatocytes. Little is known on the other activin/inhibin subunits in human liver and hepatocellular carcinoma (HCC).

**Methods:**

We studied the expression of the complete inhibin family *α*, *β*A, *β*B, *β*C, *β*E in normal liver, tumour-adjacent and HCC tissue, 12 additional organs and rodent liver. A total of 16 HCC and 10 disease-free livers were analysed. Expression of inhibin subunits was determined by qRT–PCR, normalised to RNA input and by geNorm algorithm, and confirmed by immunohistochemistry.

**Results:**

Remarkably, *βA* expression was not decreased in HCC. Similarly, *βC* and *βE* exhibited no major changes. In contrast, *inhibin α*, barely detectable in normal liver, was strongly increased in tumour-adjacent liver and dramatically enhanced in HCC. *β*B was strongly enhanced in some HCC. At variance with human liver, rodent liver showed higher *inhibin α* and *βC* expression, but *βA* was somewhat, and *βB* dramatically lower.

**Conclusions:**

Upregulation of *inhibin α* – and possibly of *βB* – may shield HCC cells from anti-proliferative effects of activin A. Dramatic variations between humans and rodents may reflect different functions of some inhibins/activins.

Liver cancer is among the leading causes of cancer deaths worldwide. It is characterised by deregulation of proliferation and apoptosis of hepatocytes and usually develops on the basis of chronic tissue inflammation leading to fibrosis and cirrhosis (Grasl-Kraupp *et al*, 2000; Drucker *et al*, 2006; Seitz and Stickel, 2006; Herzer *et al*, 2007). The underlying molecular mechanisms are barely understood (Strand *et al*, 1996; Laurent-Puig and Zucman-Rossi, 2006; Macheiner *et al*, 2006). Their elucidation is critical for the development of improved therapies for liver cancer (Schulte-Hermann *et al*, 1997; Llovet *et al*, 2003; El-Serag and Rudolph, 2007).

Activin and inhibin proteins are members of the TGF*β* superfamily, which controls cell proliferation, apoptosis, inflammation and differentiation in many cell types and organs including the liver (De Bleser *et al*, 1997; Schulte-Hermann *et al*, 1997; Rodgarkia-Dara *et al*, 2006; Deli *et al*, 2008). The mammalian inhibin family includes one *α-* (*INHA*) and four *β-*genes (*INHBA*, *INHBB*, *INHBC* and *INHBE*). Gene products dimerise to form inhibin and activin proteins. Inhibins are heterodimers consisting of the *α*-subunit and one *β*-subunit, forming inhibin A (*α*-*β*A) and inhibin B (*α*-*β*B). Activins are homodimers (activin A=*β*A–*β*A, activin B, C and E) or heterodimers (e.g., activin AB=*β*A–*β*B) of two *β*-subunits (Grusch *et al*, 2007). At the mRNA level, *INHA* is most prominently expressed in ovary, testis, adrenal and pituitary gland but reportedly not detectable in human and rodent liver (Meunier *et al*, 1988; Tuuri *et al*, 1994). Expression of *INHBA* mRNA is usually high and found in many organs including the liver. Similarly, *INHBB* mRNA has been detected in many organs but in rodent liver low or undetectable levels or only transient appearance on diverse treatments were reported (De Bleser *et al*, 1997; Kobayashi *et al*, 2000, 2002; Vejda *et al*, 2002; Jones *et al*, 2007). In contrast, *INHBC* and *INHBE* expression levels are highest in the liver, much lower in testis, adrenal and pituitary gland and almost undetectable in other organs of rodents (Fang *et al*, 1997; Vejda *et al*, 2002; Gold *et al*, 2004). In human tissues, comparisons of *INHBB*, *INHBC* and *INHBE* expression apparently have not been reported.

The resulting activin proteins may have important functions in the human liver, which are, however, still unknown except for activin A. Activin A is considered a key inhibitor of liver growth (Yasuda *et al*, 1993; Xu *et al*, 1995; Zauberman *et al*, 1997), whereas the so-called liver-specific activin C and activin E were reported to either promote (Wada *et al*, 2004, 2005) or inhibit hepatocyte proliferation (Chabicovsky *et al*, 2003; Vejda *et al*, 2003). After partial hepatectomy in mice nulled for *INHBC*, or *INHBE* and or for both no alterations in liver regeneration was observed (Lau *et al*, 2000). Studies on expression of *INHBC* and *INHBE* mRNA during liver regeneration after partial hepatectomy (Esquela *et al*, 1997; Zhang *et al*, 1997; Kogure *et al*, 1998; Lau *et al*, 2000; Gold *et al*, 2005; Takamura *et al*, 2005; Wada *et al*, 2005), or chemical injury (Kobayashi *et al*, 2002; Gold *et al*, 2003; Grusch *et al*, 2006) in rodents produced conflicting data on possible biological functions as reviewed recently (Grusch *et al*, 2007). Most of the information available were gathered in rodent liver and rarely confirmed in humans (Tuuri *et al*, 1994).

Recently, we reported that *INHBA* and *INHBE* were downregulated in chemically induced hepatocarcinogenesis in rats (Grusch *et al*, 2006). However, in patients with cirrhosis and hepatocellular carcinoma (HCC) elevated levels of serum activin A were observed and were suggested as biomarkers of liver disease (Pirisi *et al*, 2000; Yuen *et al*, 2002). Similarly, in several other malignancies deregulation of inhibin and activin at the mRNA and protein level is a common event, including tumours of endometrial (Worbs *et al*, 2007), adrenocortical (Salmenkivi *et al*, 2001; Hofland *et al*, 2006, 2007) and gonadal stromal origin (Fuller *et al*, 1999; Fine and Li, 2003; Ciris *et al*, 2004).

Consequently, in this study we analysed by qRT–PCR mRNA expression patterns of the inhibin family in human liver, HCC-adjacent fibrotic/cirrhotic tissue, and HCC. For comparison, inhibin expression patterns were also analysed in additional human tissues and in mouse and rat liver. To our knowledge, this is the first analysis of the entire inhibin family in human liver and HCC. To overcome problems resulting from uneven expression of reference genes in normal, tumour-adjacent and malignant tissue, we standardised gene expression by referring to total RNA input and by normalisation with the geNorm protocol. The results provide a distinct molecular expression portrait of the inhibin gene family in disease-free liver, HCC, other organs and rodent liver. Unexpectedly, *INHA*, barely detectable in disease-free liver, was strongly elevated in most HCC samples. *INHBB*, extremely low in rat liver, was highly expressed in human liver and further upregulated in most HCC. These results provide important new clues to functions of the inhibin/activin family in liver and HCC.

## Materials and methods

### Tissue samples

A total of 16 HCC (tumour, T) and tumour-adjacent (non-tumour, NT) liver samples, plus 5 normal (N) liver specimens were obtained from patients of the General Hospital, Vienna, in the Austrian ‘Gen-Au Programme’ (Macheiner *et al*, 2006; Sagmeister *et al*, 2008). Patient information's are listed in Table 1. Written informed consent was obtained from each patient. Additionally, 5 RNA samples were purchased, providing overall 10 normal liver specimens.

All cancer samples contained at least 80% tumour cells as shown by histology. In all, 6 tumours were classified as stage I, 7 as stage II, 1 as stage III and 2 as stage IV, and histological grading was 2 HCC-1, 13 HCC-2 and 1 HCC-3, according to AJCC/UICC standards and (Edmondson and Steiner, 1954). Hepatocellular carcinoma-adjacent tissue was cirrhotic in seven cases and fibrotic in nine cases. Human RNA from normal liver and 12 additional organs was from BioCat (Heidelberg, Germany), Clontech (Mountain View, CA, USA) and Stratagene/Agilent Genomics (Santa Clara, CA, USA). Liver samples from C57BL/6 mice and Wistar rats were obtained as described (Grusch *et al*, 2006).

In total, 5 out of 16 samples described above were available for immunhistochemical analyses. In addition, a commercially available HCC tissue array (Biochain, Hayward, CA, USA) was used. This array contains 16 pairs of tumour and adjacent tissue sections (1 stage I and 15 stage II tumours). A human ovarian granulosa cell tumour (Biogenex , Fremont, CA, USA) served as positive control for inhibin *α*.

### RNA isolation

Total RNA was isolated using TRIzol reagent (Invitrogen/Life Technologies, Carlsbad, CA, USA) according to the manufacturer's instructions. Tissue samples were prepared using a homogeniser (Bertin Technologies, Montigny-le-Bretonneux, France). High quality of RNA samples were carefully controlled on GenQuant (Pharmacia/GE Healthcare Life Science, Stockholm, Sweden). All samples of RNA were protein free as indicated by absorbance ratio 260/280 nm of 1.8–2.1. The mean error of triplicate measurements of RNA concentration of all samples (*n*=42) was 8.0%. The integrity of all RNA samples was confirmed electrophoretically on 1.5% agarose gel (data not shown).

### Reverse transcription and quantitative real-time PCR

Two micrograms of total RNA was reverse transcribed with MMLV RevertAid (Fermentas/Thermo Scientific, St Leon-Roth, Germany) resulting in 100 *μ*l cDNA solution. In all, 2 *μ*l cDNA solution was used as template for each PCR. Real-time analysis was performed with TaqMan Universal PCR Master Mix (Applied Biosystems/Life Technologies, Carlsbad, CA, USA) on an ABI Prism 7000 Sequence Detection System (Applied Biosystems/Life Technologies) running the following protocol: initial 50 °C for 2 min and 95 °C for 10 min, and 40 repeats of 95 °C for 15 s, 60 °C for 1 min. Taqman assays used in this study are listed in Supplementary Table 1. All samples were analysed in duplicate and in at least two independent real-time PCR runs. The intra-assay mean error was 0.16% and the inter-assay mean error 1.24%.

### Calculation of expression data

Original (raw) cycle threshold values *C*_t_ refer to 100 ng of total high quality RNA input (designated ‘normalised to RNA input’). They were transformed by the following procedures. For each gene, the median *C*_t_ value of data from normal liver samples (*n*=10) was used as calibrator. Each single sample *C*_t_ value was logarithmically transformed, calculated by the formula 2^−(calibrator–sample)^, and expressed as fold change *vs* the median of disease-free liver *C*_t_. For normalisation with reference genes, samples were transformed as described (Vandesompele *et al*, 2002). As expression of standard reference genes in normal and malignant liver showed large variations, we determined the most stable one using geNorm applet. For normalisation, the transformed data of inhibin genes were divided by the respective normalisation factors (NFs). The following genes were used for normalisation: for N samples *B2M*, *ACTB* and *HPRT*, for NT and T samples *GAPDH*, *HPRT* and *TBP* (additional information in Supplementary Figure 1). Subsequently, data from NT and T samples were normalised by using NF. The median of normalised data from disease-free liver samples was used as calibrator and set 1. Two different sets of NF were obtained by either assuming NT and T samples as one group, referred to as ‘NF jointly’, or as separate subgroups NT and T, referred to as ‘NF separately’. Changes in expression levels in NT and T from HCC patient's normalised by geNorm or to RNA input are compared in Table 2.

For species and tissue comparisons, *C*_t_ values normalised to RNA input were used. The results from the lowest expressing tissues were set 1 after logarithmical transformation. Results from all other samples were expressed as fold change.

### Statistical analysis

All statistical analyses were performed using GraphPad Prism 4.0 for Windows (http://www.graphpad.com). Data of all three groups (N, NT and T) were analysed by Kruskal–Wallis test. Significance of differences between medians of non-paired groups (N and NT, N and T) was checked using the non-parametric Mann–Whitney *U*-tests. Results from paired NT and T samples were analysed by the non-parametric Wilcoxon matched pair test. Correlation between gene expression levels and ratios was checked by the Spearman ranked test. All tests were performed as two-tailed and statistical significance was assumed at *P*<0.05.

### Immunohistochemistry

Histological staining was performed as previously described (Grusch *et al*, 2006). In brief, tissue samples were fixed in 4% buffered formalin, embedded in paraffin, 2 *μ*m sections were deparaffinised and antigen-retrieval was done by heating in 0.01 M citrate buffer, pH 6.0. Sections were incubated overnight at 4 °C in 0.1% BSA/PBS with primary antibodies against inhibin *α*-subunit (clone R1, LabVision/Neomarkers/Thermo Scientific, Fremont, CA, USA) diluted 1 : 20 and activin/inhibin *β*B subunit (R&D Systems, Minneapolis, MN, USA) diluted 1 : 100. Then, sections were washed in PBS with 0.5% Tween 20 and incubated with HRP-coupled secondary anti-mouse antibody (Dako, Glostrup, Denmark) diluted 1 : 200. DAB was used as chromogen to detect peroxidase activity. Sections were counterstained with haematoxylin and mounted in Dako mounting medium (Merck, Darmstadt, Germany).

Rabbit polyclonal activin/inhibin *β*E subunit antibody was kindly provided by W Schneider (Grusch *et al*, 2006). Staining for activin/inhibin *β*E (primary antibody, dilution 1 : 500) and activin/inhibin *β*A (Serotec, Duesseldorf, Sweden, mouse monoclonal, dilution 1 : 50) was performed as described above except that biotinylated instead of HRP-coupled secondary antibodies were used and sections subsequently incubated with streptavidin–HRP conjugates.

For staining of the tissue arrays, fixed in 10% formalin, the more sensitive Ultravision Detection System (LabVision/Thermo Scientific) was used. Antigen retrieval and the concentration of the primary antibodies were the same as described above. Activin/inhibin *β*C immunocytochemistry was not studied because of the lack of reliable antibodies. All available antibodies gave a positive response in *INHBC* knockout mice suggesting cross reactivity with other protein(s) (unpublished).

## Results

### Expression of the inhibin family in normal and tumour-adjacent liver and in HCC

**Figure 1:**
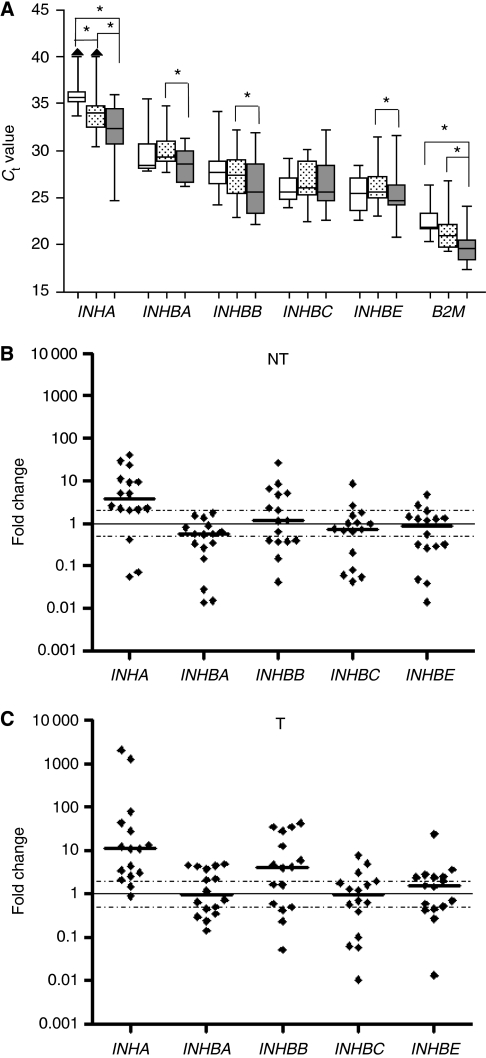
mRNA expression of inhibin genes in disease-free (N), and tumour-adjacent (NT) liver and in HCC (T). (**A**) Real-time PCR cycle threshold (*C*_t_) values normalised to RNA input are given. *C*_t_ values for *B2M* are shown for comparison. Boxes (blank, N; dotted, NT; grey; T) represent the lower and upper quartiles with medians; whiskers illustrate the 10–90 percentiles of the samples. Significant changes (*P*<0.05) are marked with brackets and asterisk. (**B** and **C**) Expression changes of individual NT or T samples related to the median of normal liver (N). Log transformed *C*_t_ values were used. Dotted lines indicate expression changes to >2-fold or <0.5-fold, considered as thresholds of biological relevance. *INHA* levels in three N and one NT samples were undetectable and for calculations were set at the detection limit (*C*_t_ 40).

We have previously studied *INHBA* and *INHBE* expression in 11 samples of human HCC and two disease-free livers (Grusch *et al*, 2006). We now extended our analysis to all inhibin family members and to tumour-adjacent (fibrotic or cirrhotic) tissue, and increased sample numbers (16 HCC, 10 normal liver specimens). PCR results are displayed in Figure 1. *B2M* is included here, as it was used as reference gene in the previous study.

*INHA* (*inhibin α*) mRNA, reportedly undetectable in rat liver (De Bleser *et al*, 1997), was found in 7 out of 10 normal (N) liver samples, range of *C*_t_ values 33.5–36.5, in 15 out of 16 adjacent tissues (NT), *C*_t_ 30.5–39.5, and in all HCC (T), *C*_t_ 24.5–36 (Figure 1A). Thus, *INHA* expression was strongly upregulated in NT and even more in T (significant for N–NT and N–T). Expression of the four *β* subunits was detected in all samples, *C*_t_ 20.5–35.5. No significant changes *vs* N were seen in NT or T. However, T *vs* NT in paired samples showed significant upregulation of *INHBA*, *INHBB* and *INHBE*, whereas *INHBC* expression remained unaltered.

Expression changes in individual NT and T samples *vs* the median of normal liver are shown in Figures 1B and C, respectively. *INHA* expression varied considerably, particularly in HCC. Overall, it was increased in all but three NT samples, and in all but two T samples, resulting in mean 9- and 225-fold increases in NT and T, respectively (Table 2, left columns). Similarly, *INHBB* was upregulated in a fraction of the HCC, resulting in an overall mean 12-fold increase, while other HCC exhibited no change or even decreases. Expression of *INHBA*, *INHBC* and *INHBE* was increased in some and decreased in other HCC, overall no significant changes were noted. Expressions in paired NT and T samples from individual patients are displayed in Supplementary Figure 2. The strong upregulation in HCC of *INHA* and the weaker increases of *INHB* genes except *INHBC* are clearly seen (compare Figure 1A). The increase in *INHBE* was significant with only two normalisation methods, see below (Table 2).

Expression of *INHBB* and *INHBC* was correlated in NT (*r*=0.897, *P*=3 × 10^−6^) and in T (*r*=0.924, *P*=6 × 10^−7^). *INHA* and *INHBA* expression was correlated in NT samples (r=0.782, *P*=0.0006), but not in T. Overall, obvious correlations between expression levels of the genes studied clinical parameters such as gender, age, tumour staging, tumour grading, viral and fibrotic/cirrhotic status were not detected.

The present data showing no significant change in expression of *INHBA* and *INHBE* in HCC seem to vary with our previous study in which decreases for these two genes were reported (Grusch *et al*, 2006). These variations are most likely due to a difference in normalisation which was performed here to RNA input, but previously to *B2M*. When normalising the present results with *B2M*, downregulation from N to T appeared again, obviously a consequence of the pronounced increase in *B2M* expression in HCC. We conclude that a single reference gene may be unsuitable for normalisation of RNA expression data in human HCC.

### Normalisation by geNorm

In Figure 1, we display RNA data normalised to RNA input or data derived therefrom by logarithmical transformation according to (Tricarico *et al*, 2002). In addition, we applied a normalisation protocol based on combinations of reference genes determined by geNorm (Vandesompele *et al*, 2002), as developed for HCC (Supplementary Figure 1).

As five of the six chosen reference genes are significantly upregulated even in non-tumourous tissue from HCC patients (data not shown), the use of one combination of reference genes for N, NT and T samples together would lead to an general overestimation of gene expression level in the disease-free liver samples. Normalisation within a subgroup, like N, with an optimised reference gene combination (Supplementary Figures 1A and B for NT samples) results in reduced statistical spread of expression of inhibin genes, rather than in shift of the median expression, which varied between 0.86 and 1.22 only after normalisation for N samples (Supplementary Figure 1C). For NT and T samples, the same three reference genes were found as the most stable ones (data not shown). The stability values S calculated for NT samples alone was far below the recommended threshold of 1 (Hellemans *et al*, 2007). However, when T samples were included into the calculation *S*-value increase dramatically, reflecting deregulation of reference gene expression (data not shown). Consequently, we used two different sets of normalisation factors, ‘NF jointly’ and ‘NF separately’, compared the results with those obtained by normalisation to RNA input. Overall, results varied in a reasonable range, indicating the reliability of the three normalisation methods used. Specifically, the strongest increase in median expression was consistently found in tumour samples for *INHA* ranging from 8.6- to 10.9-fold. Significant expression changes between NT and T were observed with *INHBA* and *INHBB*, and in part for *INHBE* (Table 2).

### Inhibin protein expression in HCC

**Figure 2:**
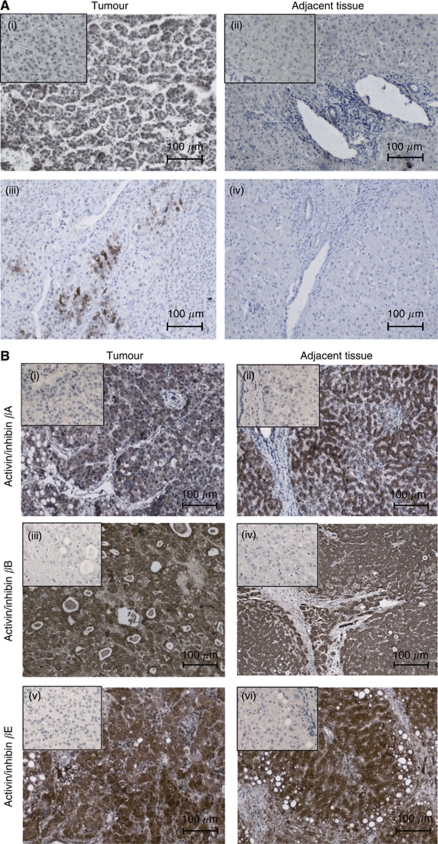
Immunohistochemical localisation of inhibin/activin proteins in HCC and tumour-adjacent liver. (**A**) Microphotographs show cytoplasmatic staining of inhibin *α* in carcinoma cells (i) and (iii), whereas hepatocytes (ii) and (iv) and non-parenchymal cells are negative. (**B**) Activin/inhibin staining for subunits *β*A, *β*B and *β*E is high in tumour and adjacent parenchymal tissue. Inlets: non-immune serum; scale bars: 100 *μ*m.

To confirm expression of inhibin genes at the protein level, we performed immunohistochemistry on a subset of the NT and T samples used for mRNA expression analysis (indicated in Supplementary Figure 2) and on a HCC tissue array from an independent patient collective. For inhibin *α*, we choose one HCC with the highest mRNA level and one moderately expressing one, as we expected to work at the detection limit. Moderate staining in almost all carcinoma cells was detected in the highly expressing HCC (Figure 2A (i)), whereas the moderately expressing tumour exhibited very intense staining in a restricted number of carcinoma cells (Figure 2A (iii)). Also in 2 of 16 samples from the tissue array, strong staining in restricted areas was detected (Supplementary Figure 3B). The adjacent tissue samples were either negative for inhibin *α* or showed a very faint parenchymal staining (Figure 2A, Supplementary Figure 3A, Supplementary Table 2).

For activin/inhibin *β*B, three representative NT samples expressing high, moderate and low mRNA were stained and the intensity of the staining correlated well with *INHBB* mRNA levels (Supplementary Figure 3B). In general, activin/inhibin *β*B staining was intense in cells from parenchymal origin in both, tumour and adjacent tissue. A semi-quantitative analysis of the tissue array data demonstrated upregulation in 7, downregulation in 4 and an unaltered expression in 5 of 16 tumour array samples (Supplementary Table 2), a similar distribution as found for mRNA expression in the independent HCC collective used for RNA analysis (Figure 1C).

Expression of activin/inhibin *β*A and *β*E protein subunits was studied in five pairs of T and NT samples. In all specimens, carcinoma cells and the parenchymal cells of the adjacent tissues were clearly positive for both proteins (Figure 2B). For both protein subunits, the staining intensities in the tissue array ranged from negative to very strong staining in tumour as well as adjacent tissues (Supplementary Table 2). With all activin/inhibin subunits, staining was restricted to carcinoma cells, hepatocytes and bile ductular cells; whereas mesenchymal cells were consistently negative. As the antibodies detect inhibin subunits, the composition of the dimeric proteins cannot be deduced.

### Inhibin gene family expression in various human tissues

**Figure 3:**
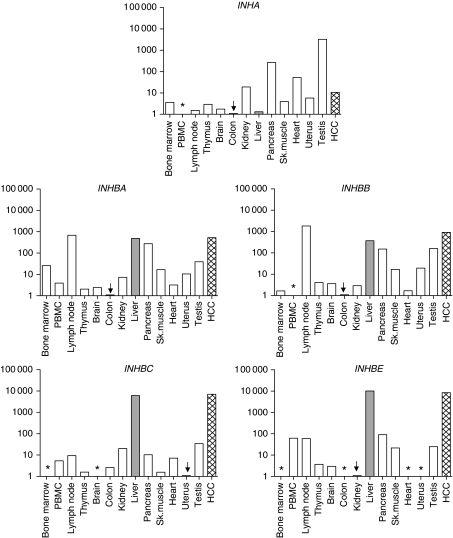
Expression of inhibin subunits in human tissues. *C*_t_ values detected in the various tissues ranged from 24.0 to 36.0 for *INHA*, for *INHBA* from 27.5 to 37.0, for *INHBB* from 24.5 to 35.5, for *INHBC* from 26.0 to 38.5 and for *INHBE* from 24.5 to 36.5. Expression below detection limits is marked by asterisks. The respective lowest expressing tissue (arrow) was used as calibrator and set 1, the expression levels of the others were depicted as fold increase in logarithmic scale. Disease-free liver is shown in grey, HCC expression levels by gridded bars. PBMC, peripheral blood mononuclear cells.

Expression of inhibin family members was analysed in 12 additional human organs and normalised to RNA input. Results including levels in normal liver and HCC are shown in Figure 3. *INHA* expression was highest in testis, as reported previously (Meunier *et al*, 1988; Tuuri *et al*, 1994), exceeding levels in HCC about 250-fold and in normal liver about 2500-fold; lowest levels were measured in colon. All four *β* subunits were highly expressed in the liver. *INHBA* and *INHBB* were detected in almost every tissue sample with a remarkably similar overall pattern. *INHBC* and *INHBE* expression were at least 100-fold higher in liver than in any other organ investigated, but nevertheless detected in 11 out of 13 and 9 out of 13 tissues, respectively. Thus, *INHBC* and *INHBE* are predominantly, but not specifically expressed in the liver as assumed previously.

### Inhibin family gene expression differs in human and rodent liver

**Figure 4:**
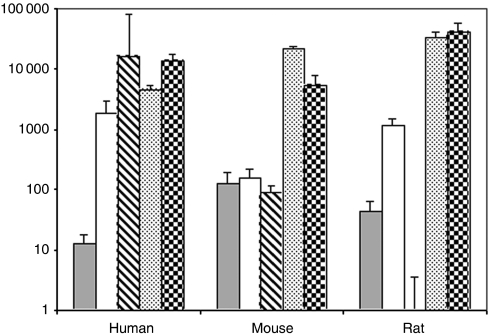
Species comparison of inhibin family expression in liver tissues. The lowest expressed gene, rat *INHBB* (*C*_t_ 37.5), was used as calibrator and set 1, the expression levels of the other genes were depicted as fold increase in logarithmic scale. Equal sensitivities of the Taqman assays were confirmed by plasmid dilution experiments (not shown). Expression ratios are depicted as following, *INHA* (grey bars), *INHBA* (open bars), *INHBB* (striped bars), *INHBC* (pointed bars) and *INHBE* (gridded bars).

For comparison, expression of the inhibin family was analysed in mouse and rat liver. To test for species differences in qRT-PCR-based *C*_t_ values, distinct plasmid dilution series were performed and revealed detectability at similar *C*_t_ levels (data not shown). As displayed in Figure 4, *INHA* expression, overall at a low level, was about 10-fold higher in rodent than in human liver. *INHBC* and *INHBE* were highly expressed in all three species, with *INHBC* levels being about 10-fold higher in rodent than in human liver. In contrast, *INHBA* and *INHBB* expression was more pronounced in human than in rodent liver, levels being 10- and 100-fold lower in mice. In rats, *INHBA* levels were only slightly lower than in humans while *INHBB* expression was extremely low, detectable only at *C*_t_ 37.5. Taken together, there are profound interspecies differences in basal hepatic expression of inhibin genes.

## Discussion

The involvement of members of the TGF*β* superfamily in hepatocellular carcinogenesis is well established (Teicher, 2001; Rodgarkia-Dara *et al*, 2006), but the individual players and their functions and mechanisms of action are still largely unknown. Here, we analysed the expression of the complete inhibin family in human liver and HCC, other human organs and rodent liver.

Care was taken to assure the validity of comparisons between liver and HCC as well as between different tissues or species. As also reported by several groups the usual reference genes are not stably expressed during stages of cancer development (Blanquicett *et al*, 2002; Ohl *et al*, 2005; Waxman and Wurmbach, 2007). The use of a single reference gene for normalisation of RNA data may lead to unreliable or even wrong conclusions. We suggest an adapted normalisation approach especially for comparison of disease-free liver with fibrothic/cirrhotic liver and HCC. Here, we show that these normalisation procedures yield similar results as normalisation to RNA input of carefully controlled quality. This similarity confirms the overall validity of the results. In agreement with other authors (Tricarico *et al*, 2002; Caradec *et al*, 2010), we conclude that normalisation to RNA input provides reliable results. However, when studying new systems validity may be rechecked with normalisation protocols such as geNorm.

We also confirmed the validity of interspecies comparison of inhibin expression in the liver (see Results), which is supported by earlier studies reporting weak or undetectable expression of *INHA* and *INHBB* in rat liver by northern or RNA protection assays (De Bleser *et al*, 1997; Kobayashi *et al*, 2000; Vejda *et al*, 2002). Overall, our study revealed remarkable interspecies differences in expression levels of some inhibins, in particular *INHA*, *INHBA* and *INHBB*. As these differences may reflect variant roles in human and rodent liver care should be taken when extrapolating results from rodents to humans. This conclusion is supported by recent work (Utoh *et al*, 2010). In a mouse xenotransplant model, human hepatocytes replaced dying mouse cells excessively resulting in liver growth to three times the normal size, while rat hepatocytes terminated growth at normal liver size. This was explained by failure of human, but not rat hepatocytes to upregulate TGF*β* and activin A type II receptors.

Inhibin and activin A were discovered as proteins regulating the release of follicle-stimulating hormone (Ling *et al*, 1986; Vale *et al*, 1986). Since then activin A has become the best characterised member of the inhibin/activin family. Besides its function in the pituitary, it is involved in many physiological processes, including embryonic development, erythroid differentiation, fibrosis, inflammation, cell proliferation and apoptosis as reviewed recently (Rodgarkia-Dara *et al*, 2006). Induction of apoptosis by activin A was described for the first time in rat liver and primary hepatocytes (Schwall *et al*, 1993). In the following years, several studies revealed a potent growth inhibitory role of activin A in the liver (Yasuda *et al*, 1993; Hully *et al*, 1994; Kogure *et al*, 1995), and activin A and TGF*β* turned out as the two dominant hepatic growth inhibitors that counterbalance or terminate growth induction by a large number of stimulating factors.

In this study, somewhat surprisingly the expression of *INHBA* and also the protein level were not downregulated in the majority of tumour-adjacent and HCC samples studied, but even increased in several HCC. How HCC cells may escape from the proapoptotic and antiproliferative action of activin A? Activin A activity is known to be antagonised by several extracellular factors, including follistatin (Esch *et al*, 1987; Mashima *et al*, 1995), fstl3 (Tsuchida *et al*, 2000) and cripto (Gray *et al*, 2003). We described previously an upregulation of follistatin expression in rat and human liver tumours (Grusch *et al*, 2006).

Similarly, inhibin A, the heterodimer of *INHA* and *INHBA* gene products, antagonises the anti-proliferative action of activin A by competitive binding to cell surface activin type II receptors (ActRIIA/B) and inhibits downstream signalling in liver cells (Xu *et al*, 1995; Lewis *et al*, 2000; Massague, 2000). In the *inhibin α* nulled mouse, activin A serum levels were enhanced more than 10-fold, leading to apoptosis and necrosis in the liver (Matzuk *et al*, 1994). Our results revealed increases of *INHA* mRNA in most of the tumour-adjacent and HCC samples studied amounting to 9- and 225-fold increases, respectively, over disease-free liver (Figure 1, Table 2). Inhibin *α* protein also was clearly detectable, at least in some HCC (Figure 2, Supplementary Figure 3). The pronounced upregulation of *INHA* may lead to a shift from production of the activin A homodimer to the inhibin A heterodimer. These data suggest that high *INHA* expression blocks the activin A signal in HCC and also in tumour-adjacent tissue (which may contain cirrhosis, a cancer prestage), and thereby provides for, or contributes to, the growth advantage of tumour cells.

Similarly, the 12-fold mean increase in *INHBB* expression in HCC compared with normal liver may cause a shift from activin A to activin AB protein, which reportedly was less inhibitory than activin A on rat hepatocyte proliferation (Niimi *et al*, 2002). In conclusion, the enhanced expression of *INHA* or *INHBB* detected in 80% of our HCC patients may serve as alternative or additional regulators besides follistatin, fstl3 and cripto, overriding the antiproliferative and proapoptotic action of activin A.

Expression of *INHBB* in normal human liver was as high as in testis (Figure 4), one of the organs with known high expression (Roberts, 1997; Vejda *et al*, 2002). Up to now only few data on *β*B expression levels in human liver are available. In human fetal liver *INHBB* was detected by northern assay at similar levels as *INHBA*, but less expressed than in fetal testis (Tuuri *et al*, 1994). Sjoholm *et al* (2006) detected very low levels of *INHBB* by RT–PCR after normalising to cyclophilin A, whereas in a large-scale expression study on the same samples (Su *et al*, 2002) liver *INHBB* levels were higher than in pituitary, but lower than in testis (http://biogps.gnf.org/; *INHBB*; 3625_at Entrez Gene). So far, because of its low or negligible occurrence in rodent liver the role of INHBB in this organ has not been elucidated.

Interestingly, some extrahepatic tumours also show upregulation of *INHA* and/or *INHBB*. *INHBB* was upregulated in malignant endometrial tissues (Worbs *et al*, 2007) and in malignant but not in benign pheochromocytomas (Salmenkivi *et al*, 2001). Overexpression of the *INHA* subunit is pronounced in gonadal stromal tumours and even used as tumour marker (Fuller *et al*, 1999; Fine and Li, 2003; Ciris *et al*, 2004). Similarly, elevated serum levels of the inhibin *α* peptide were found in granulosa cell tumour patients (Burger *et al*, 2001). No inhibin *α* protein was detected in liver (Renshaw and Granter, 1998; Lau *et al*, 2002), but was reportedly found in 17 out of 19 HCC by immunohistochemistry (McCluggage *et al*, 1997; Vrettou *et al*, 2005). However, this finding was not confirmed on 23 HCC and suggested to be a staining artefact caused by endogenous biotin (Iezzoni *et al*, 1999). Another study classified a *INHA* positive hepatic adenocarcinoma as cholangiocarcinoma (Vrettou *et al*, 2005). Our investigation provides strong evidence for expression of *INHA* in HCC as cross-reactivity with biotin is excluded by using a biotin-free detection system and the specificity of staining was shown by incubation without antibodies or non-immune serum.

In summary, we have shown a pronounced upregulation of *INHA* and a more moderate increase in *INHBB* in most of the HCC samples analysed. Furthermore, marked differences in expression of inhibin genes between rodent and human liver were found. These new insights provide important hints to the largely unknown functions of the inhibins and activins in the liver and during hepatocarcinogenesis. Understanding how cancer cells escape from the action of activin A, a major growth inhibitor in the liver, may elucidate key dysfunctions of signalling in HCC and eventually open up new avenues to molecular therapy of this disease.

## Figures and Tables

**Table 1:** Characteristics of controls and HCC patients

	**Controls (normal liver)**	**HCC patients**
Number	10	16
*Sex*		
Female	4	6
Male	6	10
		
*Age at operation*		
Mean±s.d.	50.5±6.6	63.4±12.4
Range	45–64	43-78
		
HBV infected	0/10	0/16 (0%)
HCV infected	0/10	5/16 (31%)

Abbreviations: HBV=Hepatitis B virus; HCC=hepatocellular carcinoma; HCV=Hepatitis C virus.

**Table 2:** Changes in expression of inhibin genes in tumour-adjacent and HCC samples calculated with different normalisation methods

	**Normalised to RNA input**		**NF separately**		**NF jointly**	
	**NT**	**T**	**NT**	**T**	**NT**	**T**
*INHA*	9.0±2.9	225±148	6.2±3.3	140±83	5.1±2.1	357±291
	3.7	10.9	2.3	8.6	2.3	8.9
	Wmp^*^, MW–NT^*^, MW–T^**^		Wmp^**^, MW–T^*^		Wmp^**^, MW–T^*^	
*INHBA*	0.61±0.14	1.9±0.46	0.68±0.17	6.3±3.7	0.77±0.20	3.9±1.4
	0.56	0.97	0.55	2.3	0.63	1.8
	Wmp^*^		Wmp^**^		Wmp^**^	
*INHBB*	4.0±1.7	12±4.0	2.5±1.0	30±19	2.8±1.2	16±7.6
	1.23	4.3	0.64	3.5	0.72	4.3
	Wmp^*^		Wmp^**^		Wmp^**^	
*INHBC*	1.3±0.51	1.7±0.52	1.1±0.38	3.4±1.5	1.3±0.43	2.2±0.64
	0.73	0.98	0.71	1.3	0.81	1.4
	—		—		—	
*INHBE*	1.2±0.33	3.1±1.6	0.80±0.33	3.1±1.1	0.90±0.37	2.0±0.72
	0.94	1.7	0.40	0.76	0.46	0.65
	Wmp^*^		Wmp^**^		—	

Abbreviations: HCC=hepatocellular carcinoma; MW–NT=Mann–Whitney *U-*test comparing N and NT samples; MW–T=Mann–Whitney *U* test comparing N and T samples; N=normal; NF=normalisation factor; NT=non-tumour; T=tumour; Wmp, Wilcoxon matched pair test comparing NT and T samples.

^*^P-value <0.05; ^**^P-value <0.01.

Values are given relative to the median mRNA expression value in normal liver tissue as means±s.e.m. (first row) and medians (second row).
