# Supplementary figures and images for: Inhibin/activin expression in human and rodent liver: subunits α and βB as new players in human hepatocellular carcinoma?
Source: Br J Cancer. 2011 Mar 15;104(8):1303–12. doi: 10.1038/bjc.2011.53 (PMC3078591; doi:10.1038/bjc.2011.53)

Suppl.Figure 1

A

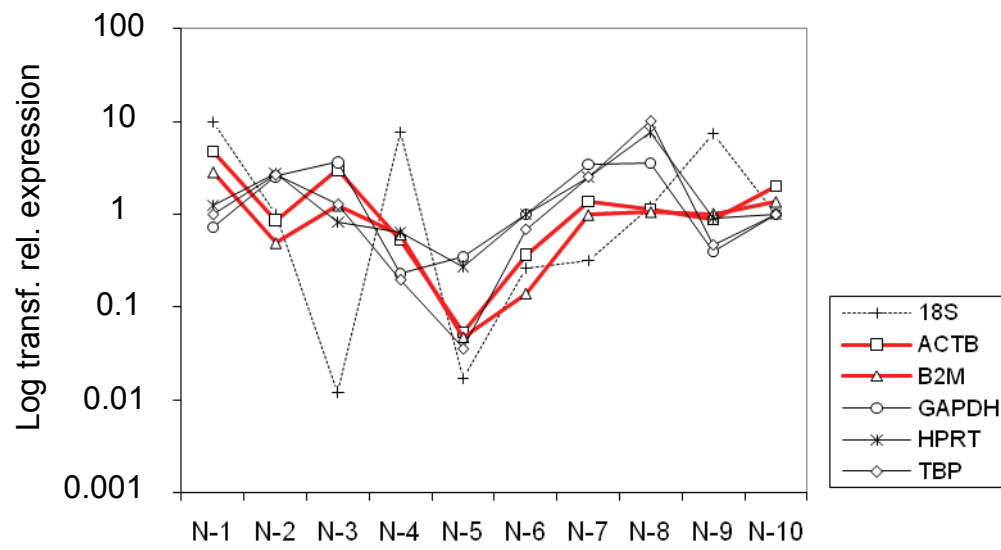

B

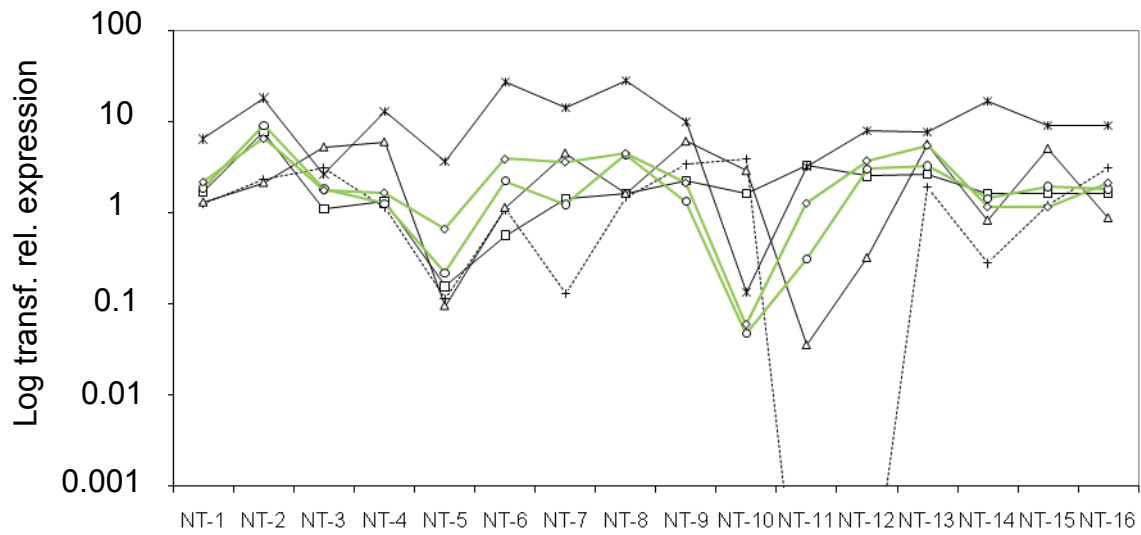

C

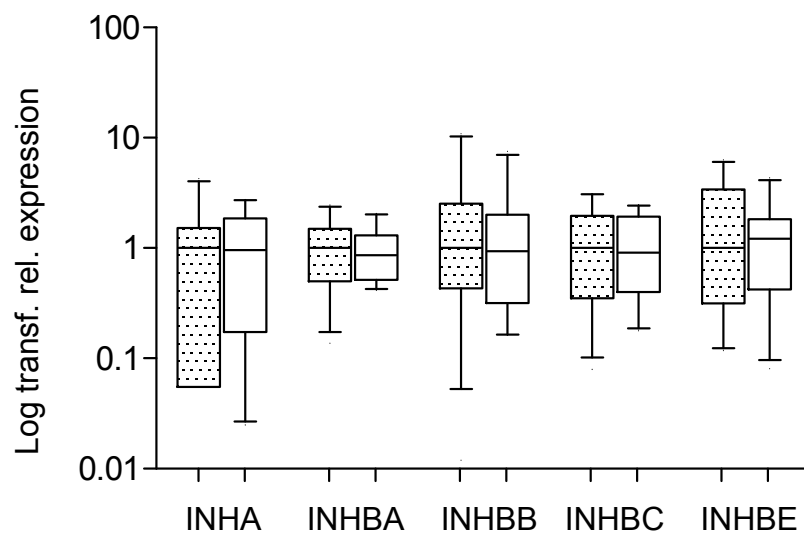

Suppl.Figure 2

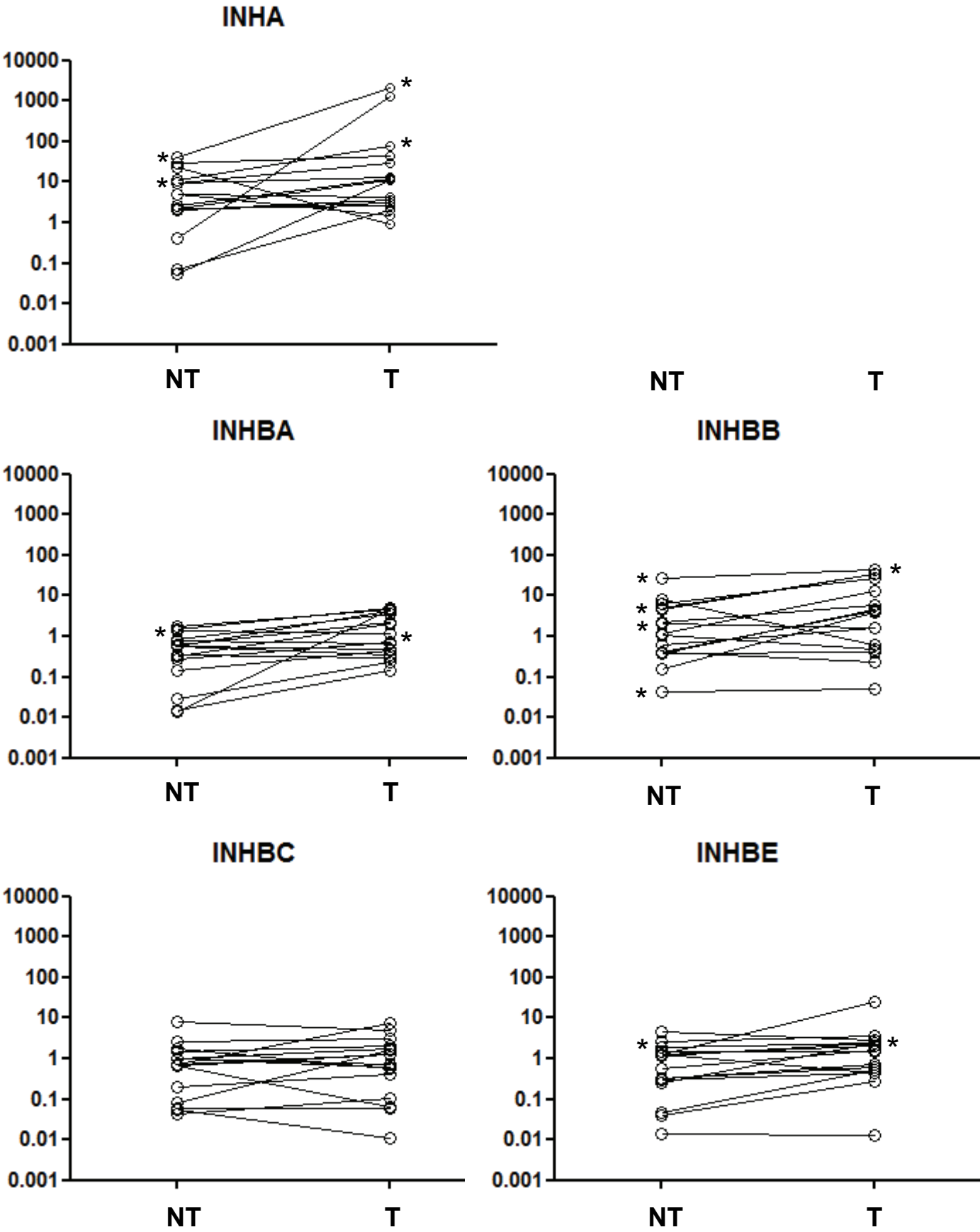

Suppl.Figure 3

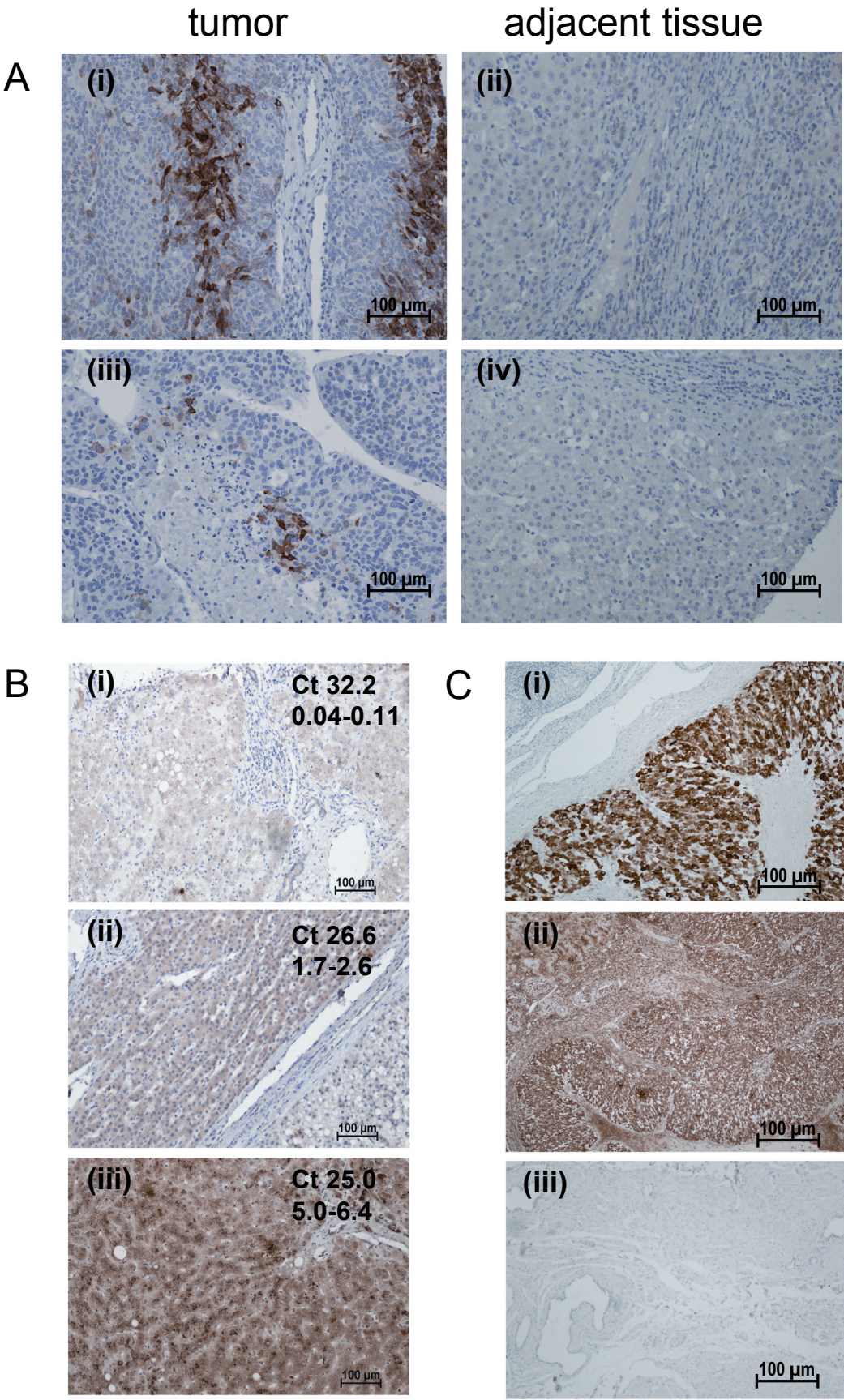

Supplement: Supplementary Figures 1–3 [file bjc201153x1.pdf]
