# Supplementary material for: Inhibin/activin expression in human and rodent liver: subunits α and βB as new players in human hepatocellular carcinoma?
Source: Br J Cancer. 2011 Mar 15;104(8):1303–12. doi: 10.1038/bjc.2011.53 (PMC3078591; doi:10.1038/bjc.2011.53)
Supplement: Supplementary Materials [file bjc201153x2.doc]

Supporting Information/ Supplementary Tables and Figures

Supplementary Table 1: Taqman® assays used for human, mouse and rat liver tissue analyses.

| Gene symbol | Gene name | Accession No. | AB number |
| --- | --- | --- | --- |
| *INHA* | Inhibin alpha | NM_002191.2 | Hs00171410_m1 |
|  |  | NM_010564.4 | Mm00439683_m1 |
|  |  | NM_012590.2 | Rn00561423_m1 |
| *INHBA* | Inhibin betaA | NM_002192.2 | Hs00170103 |
|  |  | NM_008380.1 | Mm00434339_m1 |
|  |  | NM_017128.1 | Rn00567500_m1 |
| *INHBB* | Inhibin betaB | NM_002193.2 | Hs00173582_m1 |
|  |  | NM_008381.3 | Mm01286587_m1 |
|  |  | XM_344130.3 | Rn01753772_m1 |
| *INHBC* | Inhibin betaC | NM_005538.2 | Hs00173745_m1 |
|  |  | NM_010565.3 | Mm00439684_m1 |
|  |  | NM_022614.2 | Rn00575117_m1 |
| *INHBE* | Inhibin betaE | NM_031479.3 | Hs00368884_g1 |
|  |  | NM_008382.2 | Mm00434340_g1 |
|  |  | NM_031815.2 | Rn00582653_m1 |
| *ACTB* | β-Actin | NM_001101 | Hs99999903_m1 |
| *B2M* | β-2-Microglobulin | NM_004048 | Hs99999907_m1 |
| *GAPDH* | Glycerinaldehyde-3-phosphate dehydrogenase | NM_002046 | Hs02758991_g1 |
| *HPRT1* | Hypoxanthine phosphoribosyl-transferase1 | NM_000194 | Hs99999909_m1 |
| *TBP* | TATA box binding protein | NM_003194 | Hs00427620_m1 |
| *18S* | 18s ribosomal RNA | X03205.1 | Hs99999901_s1 |

Supplementary Table 2. Semiquantitative evaluation of immunoreactions.

Immunostaining of each tumor in the tissue array was compared to the surrounding tissue by semiquantitative scores. Since staining was heterogenous in tumors and surrounding, scores were obtained as follows. The intensity of staining was categorized on an arbitrary scale of 0 to 4: 0, no immunoreaction; 0.5, pale; 1, weak; 2, intermediate; 3, strong; 4, very strong immunoreaction. The extent of staining was estimated as the percentage of parenchymal/carcinoma cells of a specific staining intensity. To avoid interobserver variability all slides were evaluated by the same person in a blinded fashion. In every case the appraisal was performed in three representative microscope fields per tissue (objective x10). The sum of the products of intensity and extent of staining was used as score of the expression level; e.g. 30% of cells grouped into staining category 0.5 and 70% into category 1: 0.3 x 0.5 = 0.15; 0.7 x 1 = 0.7; 0.15 + 0.7 = 0.85 = score. Numbers in bold indicate a difference of at least 0.3 in the staining score of the respective protein subunit in the tumor (T) vs. the corresponding surrounding (NT) tissue.

| **Array position** | **Immunostain for** | **Extent and Intensity of Immunoreaction** | | **Score for the expression level** | |
| --- | --- | --- | --- | --- | --- |
|  |  | **NT** | **T** | **NT** | **T** |
| ABC1 | alpha | 0^a^ (100)^b^ | 0(100) | 0 | 0 |
|  | Act/Inh A | 2(80);1(20) | 2(10);1(30) | **1.8** | 0.5 |
|  | Act/Inh B | 3(10);2(90) | 3(30);2(70) | 2.1 | 2.3 |
|  | Act/Inh E | 3(70);2(30) | 0.5(10);0(90) | **2.7** | 0.05 |
| ABC2 | alpha | 0(100) | 0(100) | 0 | 0 |
|  | Act/Inh A | 2(50);1(50) | 4(10);2(90) | 1.5 | **2.2** |
|  | Act/Inh B | 2(100) | 2(60);1(40) | **2** | 1.6 |
|  | Act/Inh E | 3(90);2(10) | 3(80);2(20) | 2.9 | 2.8 |
| ABC3 | alpha | 0(100) | 0(100) | 0 | 0 |
|  | Act/Inh A | 2(10);1(90) | 1(20);0(80) | **1.1** | 0.2 |
|  | Act/Inh B | 2(60);1(40) | 3(30);1(70) | 1.6 | 1.6 |
|  | Act/Inh E | 1(90);0.5(10) | 2(10);0(90) | **0.95** | 0.2 |
| ABC4 | alpha | 0.5 (100) | 0 (100) | **0.5** | 0 |
|  | Act/Inh A | 1(20);0.5(80) | 3(20);0.5(80) | 0.6 | **1** |
|  | Act/Inh B | 3(20);2(80) | 1(20);0.5(80) | **2.2** | 0.6 |
|  | Act/Inh E | 3(20);2(30);1(50) | 3(40);2(20);0.5(40) | 1.7 | 1.8 |
| ABC5 | alpha | 0 (100) | 0(100) | 0 | 0 |
|  | Act/Inh A | 0.5(80);0(20) | 1(20);0.5(40);0(40) | 0.4 | 0.3 |
|  | Act/Inh B | 3(10);2(90) | 3(80);2(20) | 2.1 | **2.8** |
|  | Act/Inh E | 2(30);1(70) | 0.5(70);0(30) | **1.3** | 0.35 |
| ABC6 | alpha | 0.5(80);0 (20) | 0(100) | **0.4** | 0 |
|  | Act/Inh A | 2(40);1(60) | 0.5(80);0(20) | **1.4** | 0.4 |
|  | Act/Inh B | 3(40);2(60) | 3(100) | 2.4 | **3** |
|  | Act/Inh E | 2(50);1(50) | 1(50);0.5(50) | **1.75** | 0.75 |
| ABC7 | alpha | 0(100) | 0(100) | 0 | 0 |
|  | Act/Inh A | 4(20);3(40);1(40) | 1(20);0.5(30) | **2.4** | 0.35 |
|  | Act/Inh B | 4(20);3(80) | 3(100) | 3.2 | 3 |
|  | Act/Inh E | 2(80);1(20) | 4(10);3(70);2(20) | 1.8 | **2.9** |
| ABC8 | alpha | 0(100) | 0(100) | 0 | 0 |
|  | Act/Inh A | 2(20);1(60) | 2(10);1(20) | **1** | 0.4 |
|  | Act/Inh B | 3(100) | 3(100) | 3 | 3 |
|  | Act/Inh E | n.a.^c^ | 4(10);3(90) | n.a. | 3.1 |
| DEF1 | alpha | 0 (100) | 4(5);0(95) | 0 | 0.2 |
|  | Act/Inh A | 1(10);0.5(90) | 3(30);1(40);0(30) | 0.55 | **1.3** |
|  | Act/Inh B | 4(70);3(30) | 3(80);2(20) | **3.7** | 2.8 |
|  | Act/Inh E | 3(100) | 0.5(50);0(50) | **3** | 0.25 |
| DEF2 | alpha | 0.5(100) | 0(100) | **0.5** | 0 |
|  | Act/Inh A | 1(100) | 2(10);1(60);0(30) | 1 | 0.8 |
|  | Act/Inh B | 3(20);2(80) | 3(50);2(50) | 2.2 | **2.5** |
|  | Act/Inh E | 3(90);2(10) | 2(40);0.5(60) | **2.9** | 1.1 |
| DEF3 | alpha | 0 (100) | 0(100) | 0 | 0 |
|  | Act/Inh A | 2(5);1(95) | 2(30);1(70) | 1.05 | 1.3 |
|  | Act/Inh B | 3(30);1(70) | 3(100) | 1.6 | **3** |
|  | Act/Inh E | 2(40);1(60) | 3(20);2(80) | 1.4 | **2.2** |
| DEF4 | alpha | 0 (100) | 0 (100) | 0 | 0 |
|  | Act/Inh A | 3(10);2(30);1(60) | 2(10);1(80);0(10) | **1.5** | 1 |
|  | Act/Inh B | 3(10);2(90) | 2(100) | 2.1 | 2 |
|  | Act/Inh E | 2(20);1(80) | 3(10);0.5(90) | **1.2** | 0.75 |
| DEF5 | alpha | 0 (100) | 4(20);2(20);0(60) | 0 | **1.2** |
|  | Act/Inh A | 2(30);1(70) | 3(10);1(20);0(70) | **1.3** | 0.5 |
|  | Act/Inh B | 2(100) | 3(10);2(90) | 2 | 2.1 |
|  | Act/Inh E | 0(100) | 3(40);0.5(60) | 0 | **1.5** |
| DEF6 | alpha | 0 (100) | 0(100) | 0 | 0 |
|  | Act/Inh A | 0.5(100) | 1(10);0.5(50);0(40) | 0.5 | 0.35 |
|  | Act/Inh B | 3(20);2(80) | 2(20);1(80) | **2.2** | 1.2 |
|  | Act/Inh E | 2(40);1(60) | 0(100) | **1.4** | 0 |
| DEF7 | alpha | 0(100) | 0(100) | 0 | 0 |
|  | Act/Inh A | 0(100) | 2(5);0(95) | 0 | 0.1 |
|  | Act/Inh B | 2(30);1(70) | 3(100) | 1.3 | **3** |
|  | Act/Inh E | 1(30);0(70) | 2(20);1(80) | 0.3 | **1.2** |
| DEF8 | alpha | 0 (100) | 2 (1);0(99) | 0 | 0.02 |
|  | Act/Inh A | 1(5); 0.5(95) | 2(10);0(90) | 0.52 | 0.2 |
|  | Act/Inh B | 2(10);1(90) | 2(90);1(10) | 1.1 | **1.9** |
|  | Act/Inh E | 0.5(100) | 1(10);0.5(90) | 0.5 | 0.55 |

1. intensity of the immunoreaction
2. extent of the immunoreaction
3. this sample detached during the immunohistochemical procedure

Legends to Supplementary Figure

**Supplementary Figure 1** How geNorm determines adequate reference gene combinations and the impact of normalization on the median values of disease-free liver. Depicted are logarithmically transformed C_t_ values of the reference genes analysed in disease-free (N) (**A)** and non-tumorous (NT) tissue **(B)** of each sample in the diagrams. On the x-axis the sample name is indicated. The two reference genes with highest correlation in their expression (marked in red for N and green for NT) are assumed as stably expressed by the geNorm algorithm. The dotted line marks the most unstable reference gene. geNorm calculates stability values (S) for each reference gene and sample-specific normalisation factors (NF). The calculated S values were 0.56 for N and 0.76 for NT, both below the recommended threshold of 1. However, when N and NT were grouped together, no reference gene combination resulted in an S value < 1. For calculation of NFs the inclusion of the next stably expressed reference gene is recommended, therefore we took three genes for NF calculation as indicated in material and methods. **(C)** Comparison of the median expression values of disease-free liver samples before and after geNorm normalization. Logarithmically transformed median C_t_ values of the respective genes were set to 1 and normalization with the sample-specific NFs was performed. Boxes (dotted: normalized to RNA input, open: normalized with geNorm NFs) represent the lower and upper quartiles with medians; whiskers illustrate the 10 to 90 percentiles of the samples. No significant differences between medians were observed.

**Supplementary Figure 2** mRNA expression of inhibin subunits in individual patients. Data are given as logarithmically transformed C_t_ values, where the median expression of disease-free liver is set 1. Paired samples of NT and T are connected by a line. Samples used for immunohistochemistry are marked by an asterisk *.

**Supplementary Figure 3** Immunohistochemical analysis of inhibin α and activin/inhibin βB expression. **(A)** Two out of 16 HCC samples on a microarray displayed positive staining for inhibin α protein. **(B)** Correlation of mRNA and protein for the inhibin/activin βB subunit. Representative examples of low (i), moderate (ii) and high (iii) expressing non-tumorous liver tissue are shown. The respective Real Time PCR C_t_ values and fold-expression ranges compared to disease-free liver are indicated. The respective samples are indicated in Supplementary Figure 2 by an asterisk. **(C)** Ovarian granulosa cell tumor was used as a positiv control for inhibin α (i) and inhibin/activin βB (ii). (iii) Non-immune serum was used instead of primary antibody as negative control. Scale bars: 100µm.
